# KRAS mutational analysis for colorectal cancer: Application: Pharmacogenomic

**DOI:** 10.1371/currents.RRN1175

**Published:** 2010-09-02

**Authors:** Grace Wang, Robin K. Kelley, GAPPNet

**Affiliations:** ^*^Center for Translational and Policy Research on Personalized Medicine (TRANSPERS), University of California, San Francisco and ^†^University of California, San Francisco

## Abstract

KRAS mutational analysis is a genetic test used in clinical practice for determining the status of the KRAS gene (wild type or mutant) in tumors from patients with metastatic colorectal cancer (CRC). Persons whose tumors are wild type may respond to therapies cetuximab (Erbitux) or panitumumab (Vectibix).

## **Clinical Scenario**

In patients with metastatic colorectal cancer (CRC), KRAS mutational analysis is used to determine the status of the KRAS gene (wild type or mutant) in tumor specimens.  Persons whose tumors are wild type may respond to therapies cetuximab (Erbitux) or panitumumab (Vectibix). [1]  Patients whose tumors harbor a mutation in codons 12, 13, or 61 of the KRAS gene do not benefit from cetuximab or panitumumab. Data are mixed whether the presence of a KRAS mutation in colorectal tumors is prognostic (i.e. whether it influences patient outcomes independent of treatment).  KRAS mutational analysis is also used to refine prognosis and treatment decisions in patients with non-small cell lung cancer (NSCLC) and is under investigation as a prognostic and/or predictive factor in other malignancies. 

## **Test Description**

KRAS mutational analysis is commercially available as a laboratory-developed test on tumor tissue. PCR methods are used to detect the most common mutations in codons 12, 13, and 61 of the KRAS gene in formalin fixed paraffin-embedded or frozen tumor tissue. Results are reported as positive (presence of a mutation) or negative (no mutation detected). [2]

## **Public Health Importance**

This test applies to persons with colorectal cancer.  It is estimated that 142,570 men and women (72,090 men and 70,480 women) will be diagnosed with and 51,370 men and women will die of colorectal cancer in 2010. The age-adjusted incidence rate for colorectal cancer is 47.9 per 100,000 men and women per year. Median age at diagnosis is 70 years. The overall 5-year relative survival is 65.0%, but varies depending on stage distribution. [3]

Approximately 20% of colorectal cancer diagnoses are in the distant or metastatic stage (for whom cetuximab or panitumumab may be indicated).  The median survival in patients with metastatic CRC  is less than 2 years. [3]

## **Published Reviews, Recommendations and Guidelines** 

**Systematic evidence reviews**

BlueCross BlueShield Technology Evaluation Center (BCBS TEC) published a systematic review in January 2009 based on retrospective analyses of 5 RCT and 5 single-arm studies. [2]

**Recommendations by independent group**

**Guidelines by professional groups**

National Comprehensive Cancer Network (NCCN) and the American Society of Clinical Oncology (ASCO) have issued clinical guidelines recommending *KRAS* mutational analysis on the tumors of all patients with metastatic CRC prior to prescribing cetuximab or panitumumab. [4] [5] [6]  

## **Evidence Overview** 

***Analytic Validity*:**

KRAS gene mutation analysis is commercially available through several labs. However, the labs themselves have not provided information on analytic performance. [2]

A recent, industry sponsored study compared different KRAS testing assays from 5 labs (Agencourt, Gentris, Genzyme, HistoGeneX, and Invitek) against the Amgen DNA Sequencing Laboratory direct sequencing assay. KRAS was classified as either wild type or mutant. Techniques were in agreement if both assays identified wild type or a mutant. Agreement was assessed by κ statistics. Agreement for each assay were reported as: HistoGeneX (kappa=0.95), Genzyme (kappa=0.94), Agencourt (kappa=0.94), Gentris (kappa=0.75), and Invitek (kappa=0.13). [7]

***Clinical Validity in Metastatic Colorectal Cancer***:****

Retrospective, subset analyses of tumor tissue samples from small clinical trials have demonstrated that tumor KRAS gene mutations are associated with lack of response to both of the EGFR-targeted monoclonal antibodies approved for use in colorectal cancer, cetuximab and panitumumab. [8] [9] [10] [11] [12] [13] [14] [15] [16] [17] [18] [19] [20] [21] [22] [23] [24] [25] [26] [27] [28] [29] [30] [31] [32] [33] [34]  The strength of this association has been substantiated in retrospective analyses of patients treated in six, large randomized studies. [35] [36] [37] [38] [39] [40]

A review of 8 studies (306 of 817 patients with tumors mutant for the KRAS gene) conducted by Linardou et al found that KRAS mutations were significantly associated with an absence of response to anti-EGFR monoclonal-antibody-based treatments (sensitivity=0.47 [0.43-0.52]; specificity=0.93 [0.83-0.97]; +LR=6.82; -LR=0.57). [41]

A recent meta-analysis of 22 studies including persons with metastatic colorectal cancer treated with cetuximab found that progression free and overall survival in persons with wildtype KRAS tumors was better compared to persons with mutated KRAS tumors. [42]

The data are mixed whether KRAS mutation is indicative of worse prognosis independent of therapy.  At present, KRAS mutational analysis is not recommended for risk assessment.

***Clinical Utility*:**

NCCN and BCBS TEC conclude that tumor testing for KRAS mutation offers clinical utility, and testing is available outside of research settings in clinical practice.  However, we did not identify studies reporting physician and patient acceptance or population-based health outcomes data from use in clinical practice.

## **Links**

The lab-based tests are regulated under the Clinical Laboratory Improvement Amendments (CLIA), and FDA premarket approval is not required. [2]

## **Acknowledgments** 

## **Funding information**

This project was funded in part by the National Cancer Institute, grant # P01CA130818.

## **Competing interests**

The authors have declared that no competing interests exist.
